# The syndromic triad of COVID-19, type 2 diabetes, and malnutrition

**DOI:** 10.3389/fnut.2023.1122203

**Published:** 2023-02-21

**Authors:** Jeffrey I. Mechanick, Elena A. Christofides, Albert E. Marchetti, Kristin K. Hoddy, Jim Joachim, Refaat Hegazi, Osama Hamdy

**Affiliations:** ^1^The Wiener Cardiovascular Institute/Marie-Josée and Henry R. Kravis Center for Cardiovascular Health at Mount Sinai Heart, New York, NY, United States; ^2^Icahn School of Medicine at Mount Sinai, New York, NY, United States; ^3^Endocrinology Associates, Inc., Columbus, OH, United States; ^4^Medical Education and Research Alliance (Med-ERA, Inc.), New York, NY, United States; ^5^Rutgers New Jersey Medical School, Newark, NJ, United States; ^6^Novo Nordisk Inc., Plainsboro Township, NJ, United States; ^7^Internal Medicine and Medical Nutrition, San Diego, CA, United States; ^8^Abbott Nutrition, Columbus, OH, United States; ^9^Joslin Diabetes Center, Boston, MA, United States; ^10^Harvard Medical School, Boston, MA, United States

**Keywords:** cardiometabolic, cardiometabolic-based chronic disease, coronavirus, COVID-19, COVID-related cardiometabolic syndrome, malnutrition, SARS-CoV-2, type 2 diabetes

## Abstract

The coronavirus disease 2019 (COVID-19) pandemic challenges our collective understanding of transmission, prevention, complications, and clinical management of severe acute respiratory syndrome coronavirus 2 (SARS-CoV-2) infection. Risk factors for severe infection, morbidity, and mortality are associated with age, environment, socioeconomic status, comorbidities, and interventional timing. Clinical investigations report an intriguing association of COVID-19 with diabetes mellitus and malnutrition but incompletely describe the triphasic relationship, its mechanistic pathways, and potential therapeutic approaches to address each malady and their underlying metabolic disorders. This narrative review highlights common chronic disease states that interact epidemiologically and mechanistically with the COVID-19 to create a syndromic phenotype—the COVID-Related Cardiometabolic Syndrome—linking cardiometabolic-based chronic disease drivers with pre-, acute, and chronic/post-COVID-19 disease stages. Since the association of nutritional disorders with COVID-19 and cardiometabolic risk factors is well established, a syndromic triad of COVID-19, type 2 diabetes, and malnutrition is hypothesized that can direct, inform, and optimize care. In this review, each of the three edges of this network is uniquely summarized, nutritional therapies discussed, and a structure for early preventive care proposed. Concerted efforts to identify malnutrition in patients with COVID-19 and elevated metabolic risks are needed and can be followed by improved dietary management while simultaneously addressing dysglycemia-based chronic disease and malnutrition-based chronic disease.

## Introduction

The coronavirus disease 2019 (COVID-19) pandemic spread rapidly worldwide in less than a year and activated an unprecedented acceleration of medical research, revealing a new understanding of the relationships among viral infections and chronic metabolic diseases. The juxtaposition of threat and swift knowledge acquisition observed during the COVID-19 pandemic contrasts starkly with the slower rise in prevalence of chronic cardiometabolic diseases and the growing clinical knowledge of residual health risks determined over many decades (1). Of note, cardiometabolic drivers, risk factors, and resulting chronic metabolic states interact with COVID-19 to create a syndromic phenotype of hazards for disease severity, morbidity, and mortality, as well as long-term insults to quality of life, symptom burden, and socioeconomic impact. In a recent narrative review, the COVID-Related Cardiometabolic Syndrome (CIRCS) (2) was introduced based on consistent and compelling evidence linking pre-, acute, and chronic/post-COVID-19 disease stages with cardiometabolic-based chronic disease (CMBCD) (3, 4).

**FIGURE 1:**
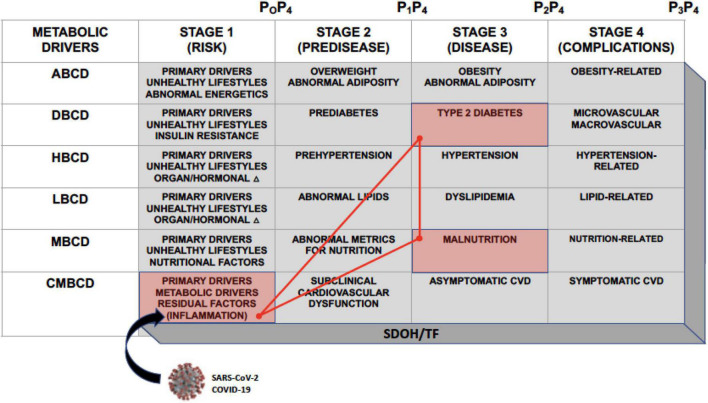
The CMBCD template. The CMBCD model comprises 3 dimensions: (1) Staged progression over time along the top row; (2) interactions among metabolic drivers culminating in CVD in the far-left column; and (3) adapting each cell in the stage x driver matrix to SDOH/TF. Prevention modalities are indicated as P_0_–P_4_. Primary drivers are genetics, environment, and behavior/lifestyle. The SARS-CoV-2 virus responsible for COVID-19 intersects with CMBCD at the level of inflammation in stage 1. This CMBCD template provides context for the novel syndromic triad of COVID-19, T2D, and malnutrition, which is depicted by the red cells and bright red triangle network. ABCD, adiposity-based chronic disease; CMBCD, cardiometabolic-based chronic disease; COVID-19, coronavirus disease 2019; CVD, cardiovascular disease; DBCD, dysglycemia-based chronic disease; HBCD, hypertension-based chronic disease; LBCD, lipid-based chronic disease; MBCD, malnutrition-based chronic disease; P_0_, primordial prevention; P_1_, primary prevention; P_2_, secondary prevention; P_3_, tertiary prevention; P_4_, quaternary prevention; SDOH, social determinants of health; TF, transcultural factors; SARS-CoV-2, severe acute respiratory syndrome coronavirus 2. Adapted from (4).

The CMBCD framework is a novel vehicle to expose opportunities for early and sustainable prevention and is comprised of three dimensions: (1) staged progression over time (1- “risk,” 2- “predisease,” 3- “disease,” and 4- “complications”); (2) multiple interacting primary (genetics, environment, and behavior/lifestyle) and secondary/metabolic (abnormal adiposity, dysglycemia, hypertension, dyslipidemia, and nutrition) drivers; and (3) social determinants of health and transcultural factors (SDOH/TF) (Figure 1) (3–5). Many of the conventional terms commonly used to describe cardiometabolic risk factors are now subsumed in driver-based chronic disease models. For instance, in adiposity-based chronic disease (ABCD), overweight is stage 2, obesity is stage 3, and obesity-related complications is stage 4 (3). In dysglycemia-based chronic disease (DBCD), insulin resistance is stage 1, prediabetes is stage 2, type 2 diabetes (T2D) is stage 3, and diabetes complications is stage 4 (3, 6). In malnutrition-based chronic disease (MBCD), which is under development, malnutrition is stage 3, and malnutrition complications is 4. The purpose of incorporating the CMBCD model into this discussion is to provide a template for understanding a specific interaction between CIRCS and nutritional status.

**FIGURE 2:**
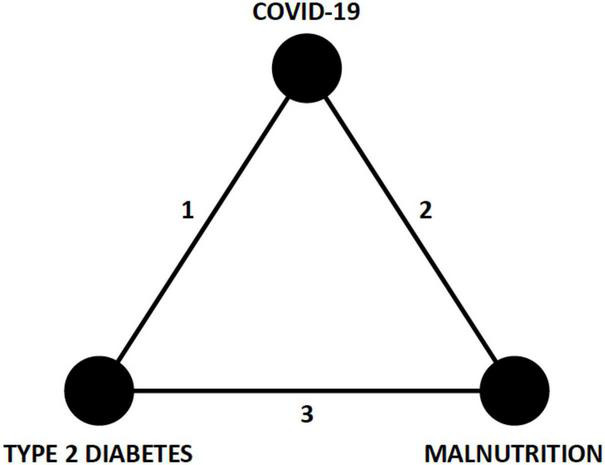
Syndromic triad of COVID-19, T2D, and malnutrition. The three edges (1, 2, and 3) of this triangle network represent epidemiological associations and pathophysiological mechanisms that connect each of the nodes (COVID-19, T2D, and malnutrition) and are discussed in the text. Recognition of this triad should prompt earlier consideration of nutritional and glycemic interventions in patients with COVID-19. COVID-19, coronavirus disease 2019; T2D, type 2 diabetes.

In a recent scoping review, various nutritional disorders are linked with pre-, acute, and chronic/post COVID-19 stages (7). A distillation of the complex interactions of CIRCS and nutrition prompts a hypothesized syndromic triad with COVID-19, T2D, and malnutrition as key inter-related disease states (Figure 2). The purpose of constructing this new triad model is to expose early opportunities for better lifestyle, glycemic, and nutritional management of patients with COVID-19. The present narrative review will summarize key epidemiological and mechanistic aspects of these networked relationships, discuss relevant nutritional therapies, and propose testable hypotheses and structure for early preventive care.

## Methodology

To guide this initiative, a virtual meeting of coauthors was held in December 2021 to establish investigative questions, objectives, and methods to support the syndromic triad concept and to plan reporting if findings were thought worthy of publication. Study populations of interest include adults who contracted COVID-19 infections that were complicated by either pre-existing or newly developed T2D and malnutrition to determine if this triad of illnesses exists and has a noticeable impact on clinical outcomes. World literature was searched for relevant articles involving the stated population using several tradition engines (PubMed, Google, Cochrane, Embase, and Science Direct) without language or geographic restrictions. The following terms, alone and in combinations, directed the searches: COVID-19, coronavirus; type 2 diabetes, malnutrition, epidemiology, mechanisms, adiposity-based chronic disease, cardiometabolic-based chronic disease, dysglycemia-base chronic disease, hypertension-based chronic disease, lipid-based chronic disease, metabolic based chronic disease, therapy, treatment, nutrition, and outcomes. Meaningful publications (181 references) among the hundreds that were identified in multiple literature searches report data regarding epidemiologies and disease mechanisms that link triad components as well as clinical information related to outcomes for studied populations. Retrieved information was assessed to confirm or deny the existence of the triad and to propose clinical care to address each component and its drivers, which was the aim of the initiative.

## Edge 1: COVID-19 and type 2 diabetes

### Epidemiology

Adults with COVID-19 are more likely to develop T2D than those with other acute upper respiratory infections (8). In an analysis of retrospective data from the US Veterans Administration (VA), there are increased risks of incident T2D and additional disease burden among patients with COVID-19 (*n* = 181,281) vs. both contemporary control patients (*n* = 4,118,911) and also historical controls (*n* = 4,286,911) without infection (9). In a study of hospitalized adults, the prevalence of diabetes is higher among those with a positive vs. negative COVID-19 test result 30 days after testing (10). The Centers for Disease Control and Prevention (CDC) reports an observed increased risk for T2D in patients <18 years of age who had COVID-19, compared to those without COVID-19 and those with pre-pandemic acute respiratory infection (11). Moreover, incident cases of pediatric T2D and severity of illness as reflected by the degree of diabetic ketoacidosis at presentation are greater during, compared with before, the pandemic (12); but also worth noting, some increase could have been associated with other issues such as delayed healthcare or supply shortages rather than infection. Pooled data from four observational studies show that SARS-CoV-2-infected patients compared with healthy controls carried a 59% higher risk of developing incident diabetes in the post-acute phase (13). However, in this study, a high degree of heterogeneity and a short follow-up period in the contributing studies (4 months) are limitations of the meta-analysis expressed by investigators.

In a single large retrospective cohort study of VA in- and outpatient men without preexisting diabetes, SARS-CoV-2 infection is associated with a higher risk of incident (120 days, OR 2.56 [95% CI 2.32–2.83]) and all time (237 days, OR 1.95 [1.80–2.12]) diabetes (14). In contrast, among women, who comprised 14% of the total VA study population, an association is not definitively established (120 days, 1.21 [0.88–1.68]; all time, 1.04 [0.82–1.31]; *p*-values were both <0.1) (14).

Many observational studies highlight compelling relationships among cardiometabolic conditions, COVID-19 infection, and severity of illness, with up to 94% of hospitalized patients presenting with at least one significant comorbidity (15–18). In a large cohort (*n* = 5,700) of hospitalized patients (17), diabetes, hypertension, and obesity are among the top comorbidities associated with COVID-19 infection, with similar patterns replicated globally in other analyses (19). Data also demonstrate a disproportionately high number of COVID-19 deaths in people with diabetes (20, 21). Likewise, obesity, with characteristic insulin resistance (22), also carries a higher risk for COVID-19 death (21).

Hyperglycemia is indicated by elevated fasting plasma glucose, post-challenge plasma glucose, and hemoglobin A1c (A1C) levels, and arises from a pancreatic β-cell defect following chronic exposure to insulin resistance. Hyperglycemia is associated with inflammation, coagulation disorders, low oxygenation, and higher risk of mortality in patients with COVID-19, compared to those without COVID-19 (6, 23). In the intensive care unit (ICU), poorly controlled diabetes with moderate-severe obesity greatly increases the risk of COVID-19-related mortality (24, 25).

Type 2 diabetes and obesity are two principal risk factors for the development of severe COVID-19 symptoms, and individuals with these comorbidities constitute a specific risk group (26). Of related interest, patients with type 1 diabetes (T1D), among other observed factors, require intensive care for COVID-19 twice as often as controls and are more likely to die (HR 2.90, 95% CI 1.66–5.47) of their COVID-19 infection (27).

Abnormal adiposity (i.e., elevated waist circumference and/or body mass index [BMI]), dysglycemia (i.e., insulin resistance or hyperglycemia [prediabetes or T2D]), elevated blood pressure (i.e., hypertension), dyslipidemia (i.e., hypertriglyceridemia and low concentration of high-density lipoprotein cholesterol), and residual risks (e.g., microalbuminuria and other features of insulin resistance) often cluster together as metabolic syndrome, which exhibits a higher odds for intensive care unit (ICU) requirement, invasive ventilation, ARDS (acute respiratory distress syndrome), and mortality compared to individual cardiometabolic risk factors (28, 29). Metabolic syndrome differs from CMBCD by only considering specific features for each metabolic driver at a particular timepoint, not as a staged progression over time based on pathophysiology, and not incorporating SDOH/TF. Aggregate cardiometabolic risk, individual risk factors, and vulnerability to severe COVID-19 each increase with age (30). Thus, cardiometabolic risk factors can be considered discretely as modifiable COVID-19 risk factors, which can be addressed with preventative approaches (i.e., “primordial” to prevent risk; “primary” to prevent disease; “secondary” to prevent disease progression; “tertiary” to prevent suffering and mortality in advanced disease; and “quaternary” to prevent overmedicalization at each disease stage).

### Mechanisms

Infection with SARS-CoV-2 appears to alter pancreatic β-cell function and consequently reduce insulin secretion, while the accompanying hypercytokinemia promotes insulin resistance (30, 31). This combination of decreased insulin secretion and sensitivity induces and then aggravates hyperglycemia. Evidence from animal models have demonstrated markers of diminished immune function in hyperglycemic states (32). In human studies, phagocytosis function is restored in subjects with T2D following an intensive glycemic control intervention combining medication, insulin, and dietary modifications provided in a clinical trial (33). In effect, a vicious cycle is created by the bidirectional relationship wherein T2D worsens COVID-19 severity and COVID-19 worsens dysglycemia.

Relative hyperglycemia for an individual at a certain time, as opposed to absolute hyperglycemia, is defined as a blood glucose concentration at a particular timepoint divided by the estimated average glucose based on a current A1C level (34). This measure, also referred to as “stress hyperglycemia ratio” (SHR) is typically measured at hospital or ICU admission to predict clinical outcomes. The SHR controls for background glycemia in patient evaluations and is also a superior marker of critical illness compared to absolute measurements. The SHR is associated with adverse outcomes for patients with moderate-to-severe COVID-19 (35), including elevated in-hospital morbidity and mortality (34).

An early investigation of patients with COVID-19 (*n* = 99; 67% men, 33% women) in Wuhan, China reports arterial and/or endocrine comorbidities in 53%, and hyperglycemia in 52% of those studied (36). Subsequently, diabetes, predominantly T2D, emerges as one of the most common and consequential comorbidities to worsen outcomes for those infected with the SARS-CoV-2 virus (37). New-onset hyperglycemia, with and without T2D, is also commonly observed with COVID-19 (38) and may result from inflammation, metabolic stress, and/or steroid therapy.

Regardless of underlying diabetes, stress-induced hyperglycemia is a well-documented occurrence during acute infections and has been observed even in mild cases of COVID-19 (35). The purported mechanisms causing hyperglycemia rely on the imbalance between insulin action and insulin secretion, and are primarily instigated by inflammation, cytokine action, neuroendocrine mechanisms, and counter-regulatory hormones (3). For a patient with T1D, the degree of hyperglycemia can be severe due to the presence of absolute insulin deficiency. The acute rise of blood glucose levels and catabolic processes can lead to diabetic ketoacidosis, a life-threatening event. In a patient with T2D, the severity of the hyperglycemia may not be a medical emergency, but the prolonged nature is associated with increased risk of cardiovascular morbidity and mortality (39). Hyperglycemia can also result in increased morbidity and mortality due to decompensation of the immune system in the face of glucotoxicity (40).

The mechanisms of increased morbidity and mortality associated with acute or chronic hyperglycemia in diabetes are multifocal. The increased cytokines of an acute inflammatory response are known to globally blunt insulin receptor responsiveness (41). Poor insulin receptor function disproportionally allows degradation of the visceral fat compartment releasing a repertoire of proinflammatory adipokines such as leptin and adiponectin (42). The blunting of glucose uptake and insulin action by adipokines further aggravates hyperglycemia. In addition, increased inflammatory cytokines combined with proinflammatory adipokines promote the glycation of proteins, rendering them pro-adherent and prothrombotic (43). The net effect of an overabundance of these reactive glycated proteins promotes endothelial dysfunction, thrombosis, hypertension, compromised cellular function, and organ dysfunction (44).

Of particular interest in patients with SARS-CoV-2 infection is the exploitation of the angiotensin-converting enzyme (ACE)-2 receptor as an entry point into cells and initiation of infection. Patients with T2D have an overactive renin–angiotensin–aldosterone system (RAAS), with ACE-2 as a principal factor (45, 46). Upregulation of ACE-2 expression in cardiomyocytes increases susceptibility to COVID-19 in patients with T2D by facilitating SARS-CoV2 cellular entry (45, 46). Abnormal adiposity is a necessary feature of ABCD and includes unusual quantity (eutopic [including visceral fat] and ectopic [e.g., intrahepatic and peri/epicardial fat]), distribution (primary related to visceral and ectopic fat), and function (i.e., adipocyte secretome; adipokine secretion). Abnormal adiposity not only leads to inflammation, insulin resistance, DBCD, and CMBCD, but also contributes to increased RAAS activation (47). These mechanisms involve cytokine activation of multiple elements of the RAAS cascade, such as angiotensinogen and ACE, resulting in inflammatory adipokine release from fatty tissue (48). This imbalance of RAAS function can increase susceptibility to COVID-19 in patients with T2D (49).

The immunopathogenesis of COVID-19 also involves an excessive inflammatory response that can intensify into a cytokine storm in extreme cases (50, 51). Numerous inflammatory pathways are activated in this process, including facilitation of immune cell (e.g., monocytes, macrophages, neutrophils, natural killer cells, and T cells) as well as stimulation and secretion of proinflammatory cytokines (e.g., interferons, interleukins, tumor necrosis factors, and chemokines [e.g., C-base sequence chemokine ligands]) (51). In turn, a proinflammatory response recruits and activates more innate and adaptive immune cells that overstimulate the immune system, leading to massive inflammation (51). This detrimental inflammatory process can incite and exacerbate acute respiratory distress syndrome (ARDS), the leading cause of COVID-19 related mortality.

## Edge 2: COVID-19 and malnutrition

### Epidemiology

Malnutrition is the necessary and central driver of MBCD, which in turn is one of the secondary/metabolic drivers in CMBCD. The American Society for Parenteral and Enteral Nutrition (ASPEN) defines malnutrition as insufficient energy intake leading to loss of weight, muscle, and subcutaneous fat; regional or widespread fluid accumulation; and decreased strength (52). Malnutrition is typically interpreted along these somewhat narrow lines that relate to undernutrition, particularly in COVID-19 discussions, but technically the broader definition includes any abnormal interaction between dietary factors and metabolism. For instance, abnormal adiposity is a form of malnutrition (i.e., imbalance of too much dietary energy for an individual’s metabolic needs—overnutrition) and is briefly considered above in the discussions on inflammation, insulin resistance, and T2D with COVID-19. However, for the purposes of presenting the syndromic triad of COVID-19, T2D, and malnutrition, the term “malnutrition” will be based on the ASPEN definition (53). In the MBCD model, stage 1 arises through complex interactions of primary drivers (genetics, environment, and behavior/lifestyle) and defines a state of nutritional risk; MBCD stage 2 arises from progression of nutritional risks to create a phenotype characterized by abnormal metrics of nutritional status, but not yet satisfying current diagnostic criteria for malnutrition (i.e., in terms of undernutrition) (54–57) or other abnormal nutritional states, and defines a state of “pre-malnutrition;” MBCD stage 3 meets established definitions for the disease state referred to as malnutrition (54–57); and stage 4 is malnutrition-related complications (generally in terms of organ dysfunction, behaviors, and other pathophysiological abnormalities).

Population-level malnutrition is associated with increased rates of fatal COVID-19 in areas where undernutrition is commonplace (58). Moreover, nutritional status is adversely affected by acute and chronic infections, which serve as negative prognosticators, especially in institutional settings (59–61). Patients with COVID-19 are especially vulnerable to the metabolic derangements associated with malnutrition, particularly in light of the significant inflammatory response that accompanies both conditions (62–64). A high prevalence of malnutrition in a general cohort of patients with COVID-19 has been reported in prospective studies (65). For example, among elderly patients with COVID-19, the prevalence of malnutrition reaches 52.7% (66). Poor nutritional status is associated with in-hospital death among 295 patients with COVID-19, including 66 with severe illness and 41 with critical illness (67). In this study, the mortality rate is 8.47% for the total study population and 37.88% for the critically ill subgroup (67). Furthermore, despite significantly different nutritional parameters and inflammatory markers across all subgroups, patients with higher Controlling Nutritional Status (CONUT) scores and lower Geriatric Nutrition Risk and Prognostic Nutritional Indices (GNRI and PNI) have a higher risk of in-hospital mortality (67).

Coronavirus disease 2019 symptoms (e.g., anorexia, nausea, vomiting, dysphagia, bloating, abdominal pain, and diarrhea) can disrupt eating and diminish adequate food consumption. In various studies, approximately 50% of patients with COVID-19 report olfactory and gustatory dysfunction, which may contribute to loss of appetite and a subsequent reduction of nutrient intake (65, 68–70). Although malnutrition associated with COVID-19 can be overlooked during the management of critical medical issues, nutrition support for patients with COVID-19 is an essential component of care, though timing and other specifics require further empirical study.

### Mechanisms

The intersection of nutritional and cardiometabolic risk in patients with COVID-19 occurs at the level of inflammation and insulin resistance (2, 6, 7). The CMBCD model represents a range of patients who may be more susceptible to infections, including COVID-19, and could benefit from nutritional interventions to mitigate DBCD, MBCD, and CMBCD progression (53, 71–73).

Various micronutrients are known to affect host immunity and the natural history of COVID-19. Some vitamins (e.g., A and D) are direct regulators of immune-cell gene expression, while others (e.g., C and E) promote a pro-oxidant milieu to improve immunity (74). Trace elements, such as zinc, copper, and iron, can modulate susceptibility to respiratory infections (74). Also, phytonutrients (e.g., berberine, curcumin, epigallocatechin gallate, genistein, resveratrol, and sulforaphane) can activate nuclear factor (erythroid-derived 2)–like 2 antioxidant transcription factor, thought to be an important mechanism in COVID-19 pathogenesis (75). Dietary fiber, a critically important component of healthy diets, is fermented into short-chain fatty acids in the intestine and can also mount significant anti-inflammatory effects (76). The net message is that all populations require a healthy eating pattern to control weight and ABCD, prevent DBCD/MBCD/CMBCD progression, and optimize immunity before, during, and after COVID-19 (2, 6, 7, 77).

The co-existence of undernutrition with micronutrient deficiencies is associated with COVID-19 and its sequelae. The effects are compounded by a disrupted sense of smell and taste, food insecurity, and social distancing that disrupts normal lifestyle behavior and leads to unhealthy eating patterns, physical inactivity, and routine change that can affect micronutrient intake (78–81). In some patients, COVID-19 also involves the gastrointestinal tract causing nausea, vomiting, and diarrhea, which further contributes to MBCD staged progression (82). In general, patients with cough, pneumonia, respiratory failure, and immune-neuroendocrine axis activation *via* a stress response to acute or chronic illness have an impaired ability to maintain adequate nourishment (83). Put another way, MBCD and other CMBCD drivers (especially ABCD and DBCD) can sufficiently alter the immune response so that prevention and treatment are compromised, and the progression of COVID-19 results in more severe disease.

Patients hospitalized with COVID-19 are at higher nutrition risk (84). Nutritional status becomes worse in patients with COVID-19 who are admitted to the ICU or require artificial ventilation (84). Immobility in the hospital bed is also associated with sarcopenia, which may affect whole-body functioning in patients with COVID-19 (85). In the short-term, these body composition changes can impact susceptibility and immunological responses to SARS-CoV-2, subsequent inflammatory response, and resulting metabolic and respiratory distress. In the long-term, these body composition changes can modulate the time required for recovery, risk of ICU-acquired weakness and long-term disabilities, and mortality risk (84). Importantly, malnutrition has been shown to persist 30 days post-COVID-19 discharge (86). As such, patients with COVID-19, especially those with diabetes, may require tailored medical nutrition therapy to improve short- and long-term COVID-19 outcomes (71, 72, 87, 88).

## Edge 3: Malnutrition and type 2 diabetes

### Epidemiology

Although abnormal adiposity (overnutrition) is one of the most common comorbidities of T2D, undernutrition is also commonplace, with a frequency of one in seven patients with a high BMI, based on an outpatient diabetes cohort (89). A 21.2% malnutrition rate has been observed among elderly patients with diabetes, regardless of BMI (90). Additionally, many other studies have been conducted to determine the frequency of coexisting T2D and malnutrition. Among hospitalized patients in Spain, the risk of malnutrition (52) is higher with T2D (90). The risk of malnutrition and the actual malnutrition rate were 31 and 13%, respectively, among patients with diabetes assessed in a Turkish outpatient clinic; whereas, a similar assessment among hospitalized patients revealed even higher numbers: 39% risk vs. 25% prevalence (91, 92). Taken together, these associations suggest that when T2D is complicated by malnutrition (i.e., when diet is insufficient to meet age-related requirements), clinical challenges worsen and warrant a diligent approach to nutrition support and prudent supplementation with micro- and macronutrients (93, 94).

Patients with T2D can also exhibit sarcopenia (95, 96), a degenerative condition characterized by decreased skeletal muscle mass and weakness, typically observed in elderly populations and commonly associated with neurodegeneration, inflammation, and/or malnutrition (97–102). The association between T2D and sarcopenia had been shown in community-dwelling elderly adults (OR = 1.40, 95% CI: 1.18–1.66) (103). Older adults with either diagnosed or undiagnosed T2D showed excessive loss of skeletal muscle mass compared with those without T2D (103). While those without T2D lose an average of 198 ± 10 g of their total lean mass per year, patients with T2D lose about 222 ± 29 g/year and patients with undiagnosed T2D lose around 340 ± 37 g/year (104). Generalized loss of muscle mass is observed after age 40 and estimated to be 8% per decade up to age 70 years, and 15–25% every decade afterward (105). Additionally, in patients with T2D, plus or minus sarcopenia, omega-3 fatty acid intake is reduced (2.6 vs. 3.0 g/day, respectively) (106). Sarcopenia also compromises glycemic control and contributes to lower energy expenditure and generalized weakness as patients age, amplifying nutritional imperatives (101).

While sarcopenia is commonly conceptualized as weight loss and weakness related to diminished muscle mass, obesity may also accompany the disorder (107, 108). Thus, patients with T2D may present with both a low BMI, characteristic of sarcopenia, and high body fat content, characteristic of adiposity, leading to the descriptive terminology—“sarcopenic obesity.” Diagnostic criteria often combine single or multiple assessments of sarcopenia with the quantification of systemic and central adiposity. Depending on definition and population, the prevalence of sarcopenic obesity ranges from 0 to 20% (with average prevalence rates between 5 and 10%) in numerous international studies of older adults (108, 109). Prevalence calculations are lower (3–8%) if the height-adjusted appendicular lean mass (ALM) index is used to define sarcopenia (110) rather than weight- or BMI-adjusted ALM indices (6–10%) (111). Moreover, prevalence rates of sarcopenic obesity are significantly higher (16–25%) among people 80 years of age and older or when lower quintiles of muscle mass or higher quintiles of body fat are factored into the assessments (112).

Among both inpatients and outpatients with diabetes, malnutrition is associated with a dysregulated immune system, higher risk for acute and chronic diseases, and protracted illness (113, 114). Such patients, particularly those with low lean body mass and high adiposity, consistently experience poorer outcomes in many different diseases (115). Manifestations of compromised immunity in patients with COVID-19 include lymphopenia upon admission and thrombocytopenia with leukopenia as infections worsen (116). Likewise, elevated levels of C-reactive protein and proinflammatory cytokines have been associated with increasing severity of illness and attendant nutritional risk (116, 117).

### Mechanisms

In general, patients with T2D and sarcopenia exhibit specific underlying pathophysiological mechanisms that have implications for nutritional care and lifestyle modifications. Among them are the consequences of aging, including altered physical activity and dietary patterns, as well as hormonal deficiencies, low-grade systemic inflammation, loss of protein homeostasis in muscle, mitochondrial dysfunction, and reduced quantity and function of small mononuclear satellite cells that abut muscle fibers (118–121). Hormonal deficiencies related to sarcopenia include growth hormone, testosterone, thyroid hormone, and insulin-like growth factor, all of which contribute to loss of muscle mass and subsequent physical weakness starting in midlife (122, 123). As anabolic hormonal signals decrease, catabolic signals increase *via* pro-inflammatory cytokines (tumor necrosis factor alpha [TNF-α] and possibly interleukin-6 [IL-6]), homocysteine and high-sensitive C-reactive protein levels rise, and muscle wasting accelerates (96, 122). Muscle loss, in turn, exacerbates insulin resistance, hyperglycemia and DBCD progression (102).

Changes in muscle metabolism and the diminished capability to synthesize sufficient protein to maintain muscle mass contribute to wasting syndromes (124). Over prolonged time, oxidized proteins accumulate in skeletal muscle, and accrued lipofuscin and cross-linked protein deposits are retained (119). Non-contractile dysfunctional protein replaces normal tissue and leads to the loss of muscle function and the diminished strength that characterize sarcopenia (125). Moreover, motor nerve cells that carry impulses from brain to muscle diminish with age, and movement is compromised by insufficient neurotransmission. Supportive satellite cells, normally responsive to injury or activity, fail to undergo functional differentiation and fusion with myocytes, leading to loss of contractile function (119, 122). These pathophysiological mechanisms can affect diaphragmatic muscles (126), which has significant implications for patients suffering from the syndromic triad of COVID-19, T2D, and malnutrition.

## Nutritional therapy in patients with acute COVID-19, T2D, and malnutrition

Although no unified therapeutic regimen exists for the comprehensive management of patients with the syndromic triad, physical activity and therapeutic nutrition represent two approaches that have proven merit across the triad spectrum. Persistent daily activity and dedicated exercise programs can improve glycemic regulation and decrease muscle degradation, while diets rich in protein or amino acids are helpful for patients with T2D and malnutrition (127). Diets that accentuate protein and antioxidants may combat sarcopenia by increasing muscle mass and strength *via* improved protein homeostasis and autophagy (the orderly degradation and recycling of cellular components) as well as reduced oxidative stress (120). Likewise, branched-chain amino acids, polyunsaturated fatty acids, selenium, vitamin D, and zinc can reduce oxidative stress, support mitochondrial homeostasis, and mitigate low-grade inflammation, thus suggesting their potential roles in the treatment of sarcopenia (128). To the contrary, however, a Mendelian randomization analysis shows little effect from these nutrients with the exception of a genetically high concentration of serum iron, which increased sarcopenia risk (129).

At the onset of the pandemic, limited therapeutic options existed to combat the specific problems eventually seen with COVID-19, especially infection complicated by T2D and malnutrition. Consequently, several expert groups in clinical nutrition adapted standard critical care guidelines centered on nutrition for COVID-19 (130). A comparison of approaches by ASPEN and the European Society for Parenteral and Enteral Nutrition (ESPEN) is given in Table 1 (131, 132). A brief summary of nutritional recommendations for patients with COVID-19 and critical illness includes: a blood glucose target of 6–8 mmol/L (106–145 mg/dL), nutrition assessments with malnutrition considerations, high-protein enteral and parenteral formulas, and up to a 50:50 ratio of fat-to-carbohydrate in patients receiving ventilatory support (132).

**TABLE 1:** Professional medical society approaches to nutrition in patients with COVID-19*.

Topic	ESPEN	ASPEN/SCCM
Use of PPE	◻	☑
Malnutrition screening	☑	◻
Malnutrition assessment	☑	☑
Nutrition intervention (in patients with malnutrition + COVID-19)	☑	◻
Feeding route	☑	☑
Indications/contraindications for EN	☑	☑
Feeding initiation	☑	☑
Feeding progression	☑	☑
Formula selection	◻	☑
Mention of specialty formulas	◻	☑
Tolerance monitoring	☑	☑
Post-mechanical ventilation considerations	☑	◻
ICU-acquired weakness	☑	◻

*ASPEN, American Society of Parenteral and Enteral Nutrition; COVID-19, coronavirus disease 2019; EN, enteral nutrition; ESPEN, European Society of Parenteral and Enteral Nutrition; ICU, intensive care unit; PPE, personal protective equipment; SCCM, society of critical care medicine.

Individualized medical nutrition therapy can include diabetes-specific nutritional formulas (DSNFs) that are commercial products designed to improve glycemic status. The DSNFs are supported by extensive clinical research using oral enteral access routes for better glycemic control in the ICU setting (131–133). Additionally, specific benefits for DSNFs are observed in a randomized clinical trial where 73% of patients are ventilated and 51% of these have diabetes upon admission. Those who receive DSNF vs. a standard enteral formula require significantly less insulin to maintain lower glycemic variability through 48 h of care (134). Likewise, a study of patients with critical illness and hyperglycemia on mechanical ventilation reports lower insulin requirements and diminished glycemic variability using a DSNF compared to a high-protein control formula (135). Patients using a DSNF also experience a lower incidence of ventilator complications (135). Interpretation and application of these findings are important for patients with COVID-19 and T2D, as hyperglycemia and glycemic variability are each associated with worse clinical outcomes (136, 137).

### Chronic/post-COVID-19

Recovery from COVID-19 also presents unique nutritional challenges related to both hospital/ICU duration and disease severity. Despite usual recommendations for increased protein intake for patients with critical illness (>1.3–1.5 g/kg/day) (132, 138), muscle loss and potential sarcopenia are still anticipated due, in part, to inactivity coupled with an inflamed hypermetabolic state (139). One Brazilian study reports a 30% decrease in rectus femoris cross-sectional area in patients with COVID-19 after just 10 days in the ICU (140). These patients may also experience post-ICU syndrome and/or dysphagia, which may adversely affect nutritional status (141–143). Special consideration for lingering COVID-19 symptoms is often necessary as 57% of COVID-19 survivors report ongoing problems through 6 months of recovery (144). In such circumstances, individualized rehabilitation efforts and conscientious diets are required to address malnutrition, sarcopenia, and/or dysphagia (145).

For patients with DBCD, particularly stage 3 T2D or stage 4 T2D with complications during prolonged recovery and rehabilitation, DSNF supplementation may be advisable as well. Research pre-dating the COVID-19 pandemic demonstrate that lower A1C values and increased body weight along with improvements in nutritional status and quality of life at 6 and 12 weeks are attainable with 2 servings/day of a high-protein DSNF in compromised older subjects (*n* = 402) with T2D and malnutrition (146). However, in a small study of enterally fed patients with T2D and unintentional weight loss, subsequent increases in weight are primarily attributed to body fat (147). Therefore, to improve body composition, rehabilitation efforts that include physical therapy or progressive resistance training should be part of multimodality care to increase muscle protein synthesis and enhance functional, metabolic, and psychological status (148, 149).

A significant knowledge gap surrounding specific micronutrient or anti-inflammatory supplementation still exists for patients with COVID-19 (7). Until more specific clinical evidence is available, expert opinions should prevail for implementing standard supplementation practices in patients with critical illness associated with COVID-19 (131). Emerging evidence suggests using vitamins, minerals, or other supportive micronutrients and standard nutrition formulas as tolerated by select patient groups.

For example, clinical practice guidelines propose administration of vitamins A, B complex, D, C, as well as selenium, zinc, and iron (132). Due to their anti-inflammatory qualities, omega-3 fatty acids are also studied in patients with critical illness and included in evidence-based guidelines (132, 150). In one study of critical illness and COVID-19, improved respiratory and renal function, along with higher 1-month survival, is noted in patients who received omega-3 fatty acid (400 mg EPA and 200 mg DHA) supplementation for 2 weeks, compared to patients receiving a standard enteral formula (151). Moreover, vitamin D and zinc gained attention for prophylaxis at the start of the pandemic (152).

In a cohort where over 50% of the sample have T2D, hospitalized patients with mild-to-moderate COVID-19 experience a faster recovery time for cough (∼3 days) and altered taste (∼5 days) when supplemented daily with 5,000 IU compared to 1,000 IU of vitamin D (153). Another study notes attenuated muscle catabolism with post-COVID-19 vitamin D supplementation of 200 IU/day for 6 weeks (154). However, the muscle retention is not reflected by improvements in physical function, which questions the adequacy of vitamin D metabolism to active 1,25-dihydroxyvitamin D in patients with critical illness and COVID-19, which could limit therapeutic potential (154, 155).

Other immunonutrients, including the amino acids glycine, arginine, and glutamine, may mitigate inflammation, protect lung and intestinal integrity during acute illness, and support muscle renewal during recovery (156). Unfortunately, the volatile status of the pandemic continues to limit clinical trials on COVID-19-specific nutrition recommendations, and some reports indicate suboptimal institutional adherence to existing guidelines (157–159). The completion of well-designed clinical trials and then creation and adoption of subsequent evidence-based guidelines is critically important for lowering mortality and shortening hospital stays (158).

## Hypotheses and structure for early preventive care: The critical role of lifestyle medicine

Patient surveys conducted during the initial 2020 quarantines and social-distancing mandates disclose disruptive changes in lifestyle and personal routines with the COVID-19 pandemic (81). In particular, routine change negatively affects diabetes self-management, delays required healthcare, and accentuates individual pandemic-associated stress (81, 160–162). The effects of widespread systemic disruptions, such as lulls in screening practices and routine medical oversight, are now more clear and prompting greater attention by healthcare professionals (163–167). Reports and qualitative assessments pointing to patient-perceived practice gaps in usual diabetes support are collectively underscoring the need for countermeasures to reverse these disruptions and restore healthy lifestyles (168). This is particularly true in contemporary multimorbidity care models that seek to manage multiple chronic disease states (e.g., chronic/post-COVID-19 + T2D + malnutrition) concurrently (168, 169). In effect, the COVID-19 pandemic draws much needed attention to comprehensive chronic disease management, creating opportunities to advance diabetes and nutrition care.

Encouragement for lifestyle modification has the potential to minimize infection risk during the COVID-19 era. For example, one prospective cohort study observes higher risk (3.5%) for COVID-19 infection and severe COVID-19 illness in participants in the lowest vs. highest quartile of diet quality (170). Specifically, crude incidence rates are 3.5% higher for COVID-19 infection in the lowest diet quality quartile compared to the highest (170). Using very low-calorie diets, which often utilize meal replacement products, and incorporating DNSFs as part of lifestyle change, support weight loss and adequate glycemic control (171, 172). Awareness of the connection between COVID-19 risk and cardiometabolic impairment presents a unique opportunity to emphasize preventive and complementary initiatives to promote better health, reduce CMBCD risk, and mitigate DBCD progression with comprehensive interventions that incorporate lifestyle modifications.

Although access to health resources is challenged during the pandemic, telehealth offers a solution with a 154% increase in usage at the beginning of the pandemic (173). Telehealth may be especially applicable to diabetes with one study reporting that 95% of diabetes-related visits are virtual during the first year of the pandemic (174), and its use in the diabetes space is associated with improved patient outcomes (175–180). As an example, a recent meta-analysis reports increased time in range by 70.74 min and a slight decrease in A1C (−0.17%) among people using continuous glucose monitors (CGMs) compared to usual care (175). This effect could stem from healthful behavior modification associated with CGM use (technological nudges and motivation) (176). The integration of telehealth stands to diminish pre-pandemic barriers to healthcare, but it is important to consider stakeholder acceptance and inclusion of vulnerable populations (181).

The creation of a new construct—the syndromic triad of COVID-19, T2D, and malnutrition—not only allows the derivation of hypotheses relating early detection and management of malnutrition with mitigation of ABCD, DBCD, MBCD, and even CMBCD progression, but also prompts clinical decision-making now centered on early implementation of healthy lifestyle change. The pragmatic value of this new triad framework is supported by the coalescing of multiple clinical imperatives (i.e., COVID-19, dysglycemia, and nutrition) into a focused comprehensive approach. Core recommendations, which will require clinical validation, include:

1. Conduct aggressive case-finding protocols for malnutrition in all patients with COVID-19 at any DBCD stage;
2. Implement current standards of care to optimize nutrition in all patients with COVID-19 at any DBCD stage who have malnutrition or are at-risk for malnutrition;
3. Clarify and manage specific DBCD stages in all patients with COVID-19 at any MBCD stage; and
4. Assign a higher risk classification to patients newly diagnosed with COVID-19 when any DBCD or MBCD stage is also present.

## Conclusion and future directions

There is an inherent association of COVID-19, T2D, and malnutrition supported by theoretical modeling, epidemiological data, and mechanistic relationships. Metabolic changes incurred by COVID-associated systemic inflammation increase the risk of dysglycemia, muscle protein catabolism, and nutritional deficiencies. Moreover, both T2D and malnutrition are risk factors of severe COVID-19. Awareness of these associations should encourage early diagnosis, prevention, and management of dysglycemia and malnutrition especially in vulnerable populations. Nutritional and lifestyle interventions aiming at optimizing glycemic control and improving nutritional status, as well as muscle health, could potentially decrease risk of COVID-19 complications. An individualized T2D-specific lifestyle and nutritional approach, and a close monitoring and management of glycemic status by experienced healthcare professionals, are essential to improve clinical outcomes for people with COVID-19.

## Author contributions

All authors listed have made a substantial, direct, and intellectual contribution to the work, and approved it for publication.
